# Iatrogenic peripheral pulmonary air embolism following intravenous contrast administration for CT pulmonary angiography: proposal of the “double bronchus sign”

**DOI:** 10.1259/bjrcr.20160097

**Published:** 2017-02-13

**Authors:** Joanna Moser, Sarah Sheard, Jaymin Patel, Charlie Sayer, Brendan Madden, Ioannis Vlahos

**Affiliations:** St George’s University Hospitals, NHS Foundation Trust and School of Medicine, London, UK

## Abstract

We present a case of iatrogenic extensive air embolism in the peripheral pulmonary arterial tree following intravenous contrast injection for a CT pulmonary angiogram performed to investigate chest pain in a 25-year-old female patient. Small volumes of iatrogenic air embolism following contrast injection are not infrequently encountered incidentally in the central vasculature (brachiocephalic veins, superior vena cava, right cardiac chambers and main pulmonary arteries). To our knowledge, however, this is the only case of extensive peripheral pulmonary arterial air embolism on CT that has been reported in the literature. Despite the extent of peripheral air, this potentially clinically significant complication was relatively inconspicuous at CT interpretation. A new radiological sign, the “double bronchus sign”, is proposed as a useful diagnostic tool. In addition to discussing the imaging features, important safety considerations and principles of immediate management, relevant to all radiologists, are addressed.

## Clinical presentation

A 25-year-old female patient complained of a 2-day history of pleuritic chest pain during an admission to the acute medical unit with newly diagnosed Addison’s disease presenting with an Addisonian crisis. The patient denied dyspnoea, cough or infective symptoms, and there was no history of trauma. She took the oral contraceptive pill but no other drugs. Clinical examination elicited a postural drop in blood pressure (98/95 mmHg on lying, 87/71 mmHg on standing) but was otherwise unremarkable. Preliminary investigations revealed a moderately raised D-dimer of 422 ng ml^–1^. Chest radiography was normal. In the absence of another identifiable cause for the chest pain, pulmonary embolus was considered as a differential diagnosis and the patient was referred for CT pulmonary angiography.

## Imaging

**Figure 1.**
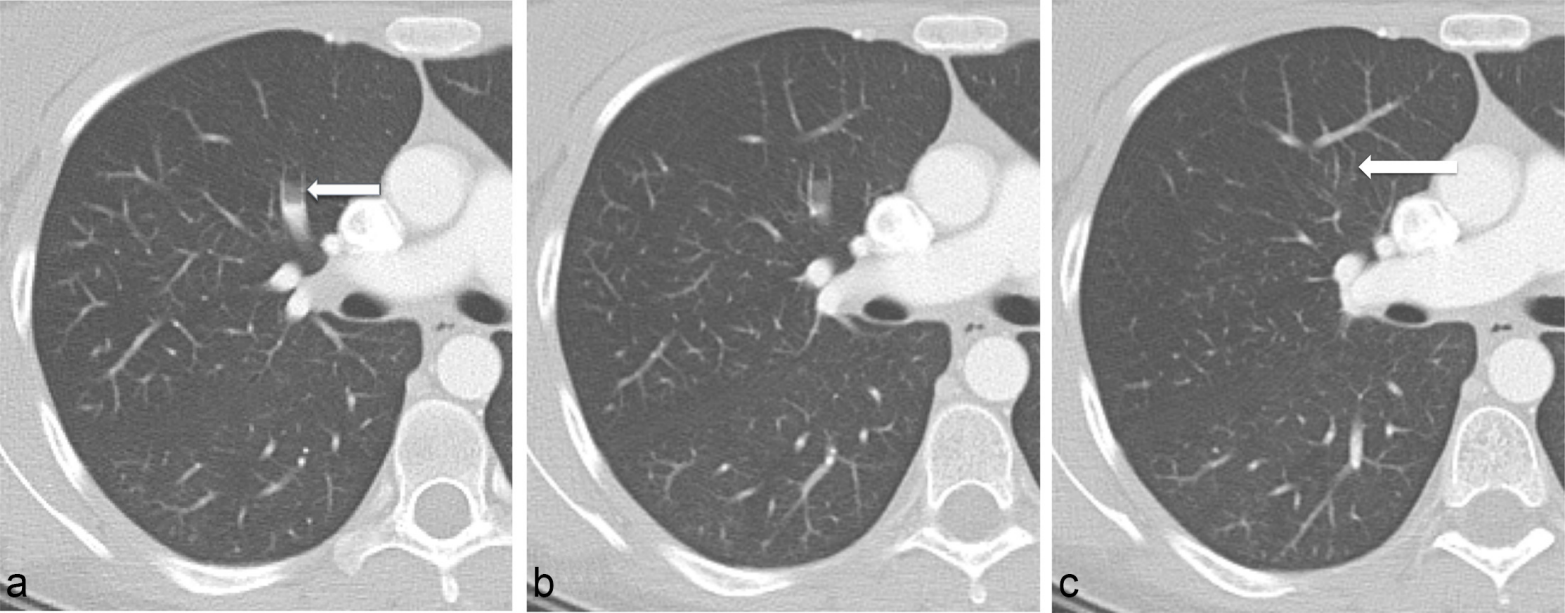
Consecutive 2.5 mm axial slices through the right upper lobe anterior segmental artery (arrows) reviewed on lung windows. Gradual antidependent fading of contrast is demonstrated peripherally (a and b), with replacement of contrast with air, in keeping with peripheral pulmonary arterial air embolus. Distally (c) the completely air-filled artery resembles a bronchus, running parallel to another bronchus: the “double bronchus sign”. This appearance was replicated in multiple antidependent segmental and subsegmental pulmonary arteries, involving all lobes.

An initial CT pulmonary angiography scan was considered non-diagnostic owing to poor contrast opacification and was immediately repeated. On the second attempt, sufficient pulmonary arterial contrast was achieved and revealed no pulmonary thromboembolic disease. On close inspection of the pulmonary arterial tree on soft tissue windows, however, there was the impression of proximal truncation of several segmental and subsegmental pulmonary arteries. Review on lung windows elicited that these vessels were not truncated but that intraluminal contrast within these vessels gradually faded peripherally and was replaced with air (Figure 1). Multiple antidependent segmental and subsegmental pulmonary arteries were affected and all lobes were involved, in keeping with extensive peripheral pulmonary arterial air embolism.

**Figure 2.**
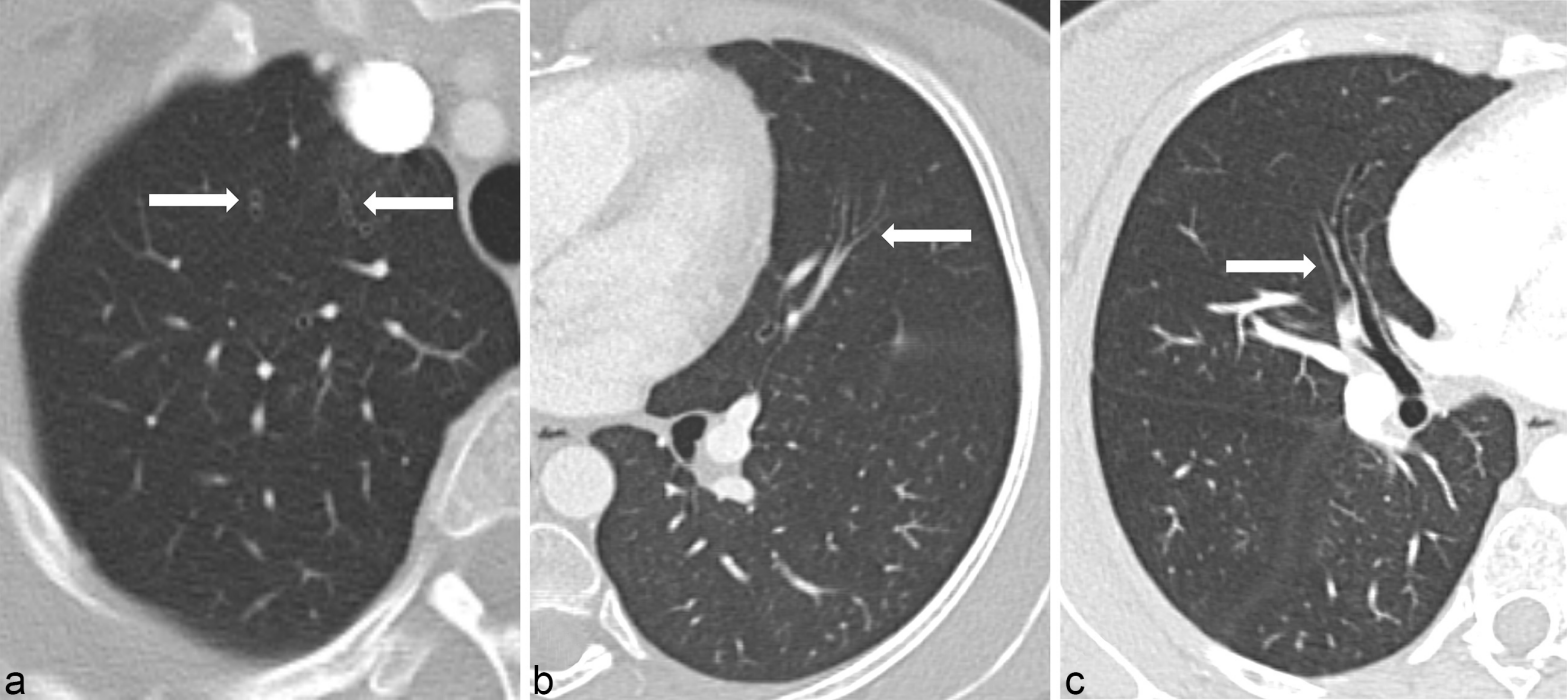
Further examples of the “double bronchus” sign (arrows) in axial section through right apex (a) and oblique reformats through left upper lobe subsegmental (b) and right upper lobe segmental (c) pulmonary arteries. Peripheral pulmonary arterial air emboli running alongside their paired bronchi give the false impression of twinned bronchi running parallel alongside each other.

The peripheral pulmonary arterial air emboli produced a CT appearance of what appeared to be twinned bronchi running parallel to and directly alongside each other. This phenomenon is not encountered in normal anatomy and was, in fact, caused by air emboli in peripheral pulmonary arteries running alongside their paired bronchi. We propose this appearance as the “double bronchus sign” (Figure 2), which has not been previously described.

**Figure 3.**
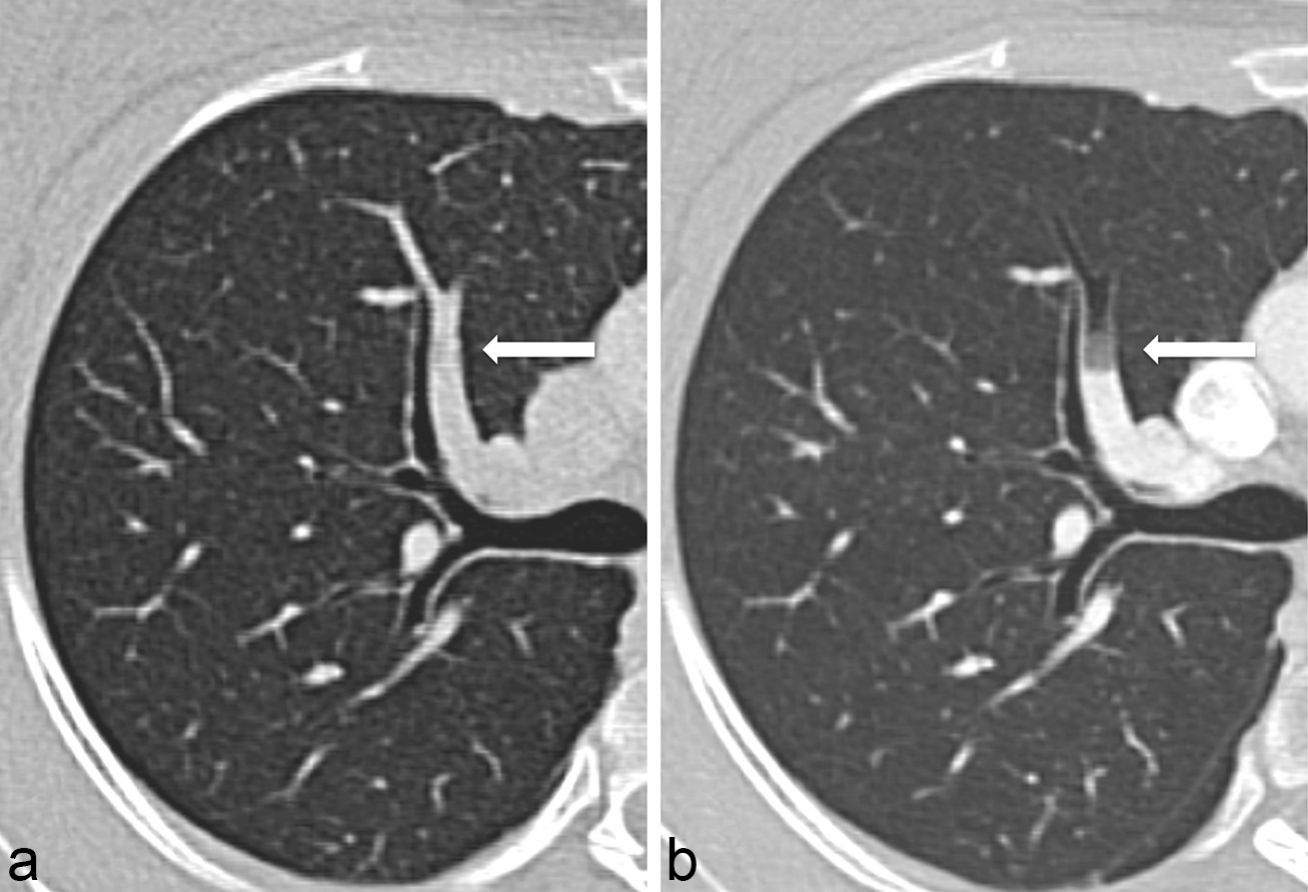
Oblique reformats through the right upper lobe anterior segmental pulmonary artery on the first acquisition (a) demonstrate no pulmonary embolus and on the second acquisition (b) clearly demonstrate peripheral pulmonary air embolus. The two acquisitions were performed in quick succession, using the same peripheral cannula, and the patient remained on the scanner between the two attempts.

Crucially, direct comparison of the two scans revealed that air embolus was only present on the second acquisition (Figure 3), confirming that the aetiology was iatrogenic and directly related to contrast injection. Both studies were performed using a power injector connected to the same peripheral cannula. No intervention, other than replacement of contrast between the two injections, had been performed between the two studies and the patient remained supine on the scanner between acquisitions. The radiographers reported that they had primed the connecting tubing to expel redundant air appropriately and there was neither detectable extravasation nor air leak.

No air was demonstrated in the brachiocephalic vein, superior vena cava, right cardiac chambers or pulmonary trunk – central sites that are not infrequently found to contain (usually inconsequential) small volumes of gas following contrast injection. There was also no detectable air in the left or right main pulmonary arteries.

No other abnormality was identified to account for the patient’s initial presentation; the lungs, pleural surfaces, cardiomediastinal structures and ribs were normal, with a normal sized pulmonary trunk noted and no CT evidence of right heart strain.

## Treatment and outcome

Despite the extensive nature of the air embolus, the patient did not experience any new symptoms or exacerbation of her dyspnoea or chest pain following the CT pulmonary angiogram. The clinical team was promptly informed of the findings and, following advice from the pulmonary hypertension team, 100% oxygen was administered and the patient was admitted for 24-h observation. She was haemodynamically stable throughout this time and maintained her oxygen saturations, with resolution of her previous chest pain, no dyspnoea and no neurological signs. An echocardiogram performed in this period demonstrated no intracardiac shunt and no evidence of pulmonary hypertension. The patient was discharged following overnight observation and was reviewed at a 6-week follow-up clinic appointment, at which point she was well and reported no further symptoms.

## Discussion

We propose the “double bronchus sign”, described above, as a helpful diagnostic aid in the diagnosis of peripheral pulmonary arterial air embolism. The appearance is somewhat subtle and could be easily overlooked if not specifically sought, particularly in the most peripheral branches. Once identified in one area, careful assessment of the rest of the pulmonary arterial tree is suggested to estimate the extent of air embolus burden. In this case, the most obvious sites of disease in the right middle and upper lobe segmental arteries were identified initially but the full extent of air embolus only became evident after more detailed interrogation of the pulmonary arterial tree.

The CT appearance of peripheral pulmonary arterial air embolism has not been previously reported in the literature, be it iatrogenic, as in this case, post-traumatic or related to decompression illness. By contrast, small volumes of venous or central air embolus following contrast injection have long been recognized and are frequently encountered in clinical practice, usually with no clinical consequence. In the largest study to have addressed this, by Groell et al,^1^ 79 of 677 (11.7% ) of patients undergoing contrast-enhanced CT of the thorax demonstrated air embolus of small to moderate volumes in the main pulmonary artery (n = 54, 8.0%), superior vena cava, right ventricle, subclavian or brachiocephalic vein, right atrium or more than one of these sites. No cases of peripheral pulmonary arterial air embolus were reported in this series. The frequency of central vascular air embolism has demonstrated no relationship to technical factors such as contrast flow rate, site and size of peripheral cannula, neither in the study by Groell et al nor in a more recent, similar study of 200 patients.^2^

Interestingly, our patient had a conspicuous lack of venous or central air embolus, both on the first and second CT pulmonary angiogram acquisitions. The reason for the peripheral distribution of air in her pulmonary arterial tree is not understood. We considered that it may have been related to a tachycardic hyperdynamic circulatory state, but this would not be a unique feature among the population of patients undergoing CT pulmonary angiography. The patient’s cardiac and pulmonary arterial anatomical configuration was normal.

While the majority of air emboli pass without clinical consequence, the potential for harm and, thus, the root of clinical concern is two-fold. First, even small volumes of air embolus can have significant clinical effects in the presence of right-to-left shunts causing paradoxical systemic emboli. This is of particular significance in patients with known intracardiac shunts or with established pulmonary disease, which may coexist with peripheral shunts undetectable on imaging. Secondly, large volumes (greater than that shown in the case we report) can additionally cause rapid onset pulmonary hypertension due to pulmonary arterial occlusion or circulatory compromise if air locks occur at the pulmonary outflow tract.^3^ This has been reported to result in sudden onset “air hunger”, dyspnoea, coughing, crushing chest pain, dizziness and a feeling of impending death.^4^ Indeed, rare cases of fatal massive air embolism through inadvertent power injection of air rather than iodinated contrast media have been reported.^5^

The immediate management of large central air embolus should be familiar to all radiologists, namely, administration of 100% surface oxygen (to create a favourable environment to decrease partial pressure of nitrogen in the air bubbles, thus reducing their size), left lateral decubitus position (to keep air in anti-dependent portions of the right heart, allowing blood flow into the right ventricular outflow tract) and lowered head, so-called “Trendelenburg” position (to direct air away from the coronary ostia and head and neck vessels). In severe cases, right heart catheterization to aspirate the air may be required.^4^ Our patient received immediate 100% oxygen to assist in reducing the volume of intra-arterial air but did not require the described positional manoeuvres in view of the paucity of central air in the venous system or cardiac chambers.

The precise cause of the air embolus in this case was never identified. It is postulated that there may have been a loose connection that was not identified at the time of scanning, but this cannot be confirmed. No prior or subsequent similar cases have been reported at our institution through use of the same power injector.

## Learning points

1. The CT appearance of peripheral pulmonary arterial air embolus has not been previously described in the literature. We propose the “double bronchus sign” as a helpful diagnostic tool in recognizing what may otherwise be a subtle imaging appearance.
2. Iatrogenic air embolus through contrast injection is a known risk of contrast injection but one that should be diminished by adequate staff training and adherence to appropriate injection protocols.
3. Recognition of the clinical and CT appearances of air embolus is important owing to the risks of paradoxical systemic emboli and, in cases of larger air volumes, rapid onset pulmonary hypertension (due to occlusion of peripheral pulmonary arteries), circulatory compromise or, in rare instances, death.
4. Radiologists should be familiar with the immediate management of large air emboli: application of 100% oxygen, left lateral decubitus, Trendelenburg positioning and consideration of right heart catheterization for aspiration. In this case of peripheral pulmonary arterial air embolus, 100% oxygen was administered and the patient was observed with no adverse clinical outcomes.

## Consent

Written informed consent for the case to be published (including accompanying images and case history) was obtained from the patient.
